# Body size of fungus-growing termites infers on the volume and density of their fungal cultivar

**DOI:** 10.1098/rsos.230126

**Published:** 2023-06-07

**Authors:** Chun-I Chiu, Jie-Hao Ou, Kuan-Chih Kuan, Chi-Yu Chen, Yin-Tse Huang, Yuwatida Sripontan, Hou-Feng Li

**Affiliations:** ^1^ Department of Entomology, National Chung Hsing University, 145 Xingda Road, Taichung 402202, Taiwan; ^2^ Department of Plant Pathology, National Chung Hsing University, 145 Xingda Road, Taichung 402202, Taiwan; ^3^ Department of Entomology and Plant Pathology, Chiang Mai University, Chiang Mai 50200, Thailand; ^4^ Innovative Agriculture Research Center, Faculty of Agriculture, Chiang Mai University, Chiang Mai 50200, Thailand; ^5^ Department of Biomedical Science and Environment Biology, Kaohsiung Medical School, 100 Shin-Chuan First Road, Kaohsiung 80708, Taiwan; ^6^ Entomology and Plant Pathology Section, Faculty of Agriculture, Khon Kaen University, Khon Kaen 40002, Thailand

**Keywords:** insect-fungus symbioses, partner choice, size-number trade-offs, niche partitioning

## Abstract

The body size of an animal plays a crucial role in determining its trophic level and position within the food web, as well as its interactions with other species. In the symbiosis between *Termitomyces* and fungus-growing termites, termites rely on nutrition of fungal nodules produced by *Termitomyces*. To understand whether the size of termites and fungal nodules are related to their partner specificity, we quantified the size of termite farmer caste, and the size and density of nodules in termite nests of four genera of fungus-growing termites, and identified their cultivated *Termitomyces* fungus species based on internal transcribed spacer regions and partial large subunit ribosomal RNA gene sequences. The results showed that the size and density of fungal nodules were different among *Termitomyces* clades and revealed a constant trade-off between size and density among clades. The nodule size of each clade has low variation and fits normal distribution, indicating that size is a stabilized trait. Moreover, we found larger termite genera cultivated *Termitomyces* with larger but less numerous nodules. Based on these results, we concluded that there is a size specificity between *Termitomyces* and fungus-growing termites, which may lead to diversification of *Termitomyces* as adaptations to different termite genera.

## 1. Introduction

Body size is an essential factor that influences the trophic positions of animals in the ecological network and their interactions with other organisms [1–4]. It is well-documented that the body size of animals is positively correlated to their positions in the trophic hierarchy [1–4]. The nutritional requirements of animals impose limits on their dietary breadth, which are further influenced by their body size. As a general trend, larger animals tend to consume prey items that are relatively larger in size [2,3,5,6]. Interestingly, positive correlations in size between symbiotic partners have also been reported in various symbiotic relationships, including parasite–host [7,8], ant–isopod [9], ant–plant [10] and pollinator–plant [11] interactions. In the context of pollinator–plant interactions, it has been observed that the size of pollinators plays a critical role in determining their host range. Specifically, larger pollinators are known to possess longer tongues, which allow them to access nectar in flowers with longer corollas or spurs [11].

The symbiosis between fungus-growing termites (Termitidae: Macrotermitinae) and their obligatory fungal symbiont, *Termitomyces* spp. (Lyophyllaceae), facilitate a major portion of woody and leaf litter decomposition in many tropical and subtropical ecosystems [12–14]. Fungus-growing termites cultivate *Termitomyces* in their nest for digestion of lignocellulose and obtaining concentrated amino acids [15–18]. In the farming system of termites, major workers are responsible for collecting woody and leaf litter in the field, while minor workers, which are relatively small-sized workers, are the caste performing farming behaviours [19]. Minor workers use their faeces to construct fungus gardens as farms for cultivating *Termitomyces* [19,20]. Termites harvest and consume the fungal nodules grown on fungus gardens, which are clusters of spores and conidiophores produced by mycelium of *Termitomyces* spp., to obtain essential amino acids and spores for inoculation [18]. The spores remaining in intestine are mixed with the plant tissues ingested by termites and are excreted with faeces of termites [21,22], then the faeces inoculated with spores are deposited on the top of fungus gardens as new fungus farms [18].

Interestingly, fungus-growing termites have a strong diversification of body size among genera. For example, *Macrotermes*, *Odontotermes* and *Microtermes* are common sympatric fungus-growing termites in Thailand [23]. Head width of soldiers of *Macrotermes* (approx. 4.31 mm) is more than double that of *Odontotermes* (approx. 1.87 mm) and quadruple that of *Microtermes* (approx. 0.85 mm) [24,25]. The body size of soldier and worker castes are correlated [26], which predicts that worker castes of different termite genera also have different sizes. The existence of size variation among termite genera suggests that they may differ in their food intake. Hypothetically, such differences in diet should also manifest in the size and number of fungal nodules associated with these species. Moreover, there may be a certain degree of size specificity between termites and their fungal symbionts belonging to the genus *Termitomyces*, which has never been examined.

In this study, we aimed to examine whether (i) each genera of fungus-growing termites cultivate *Termitomyces* spp. with a specific size and number of nodules, and (ii) larger termite genera cultivate *Termitomyces* spp. that produce larger fungal nodules. To test these hypotheses, we excavated 43 termite nests from four genera, including *Macrotermes*, *Odontotermes*, *Ancistrotermes* and *Microtermes*, measured the head width of minor workers, collected fungus gardens from nests for quantifying the diameter and density of fungal nodules produced by *Termitomyces*, and identified the fungal nodules using internal transcribed spacer (ITS) regions and partial large subunit ribosomal RNA gene (LSU) sequences.

## 2. Methods

### 2.1. Fungus gardens and termites

**Figure 1.**
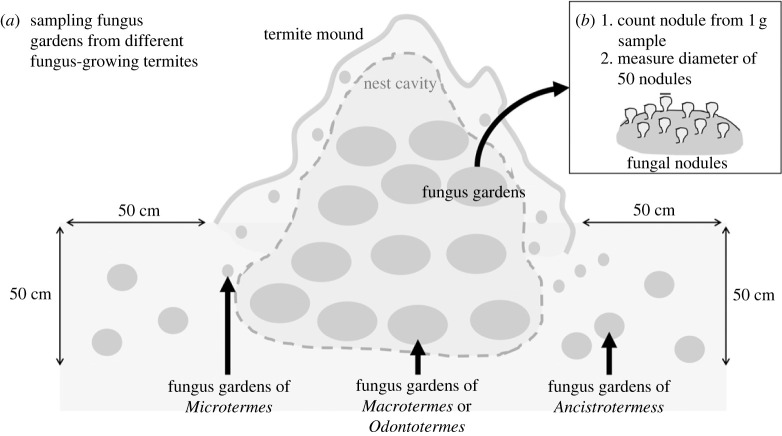
Sampling of termite fungus gardens and quantification of size and density of fungal nodule. (*a*) Fungus gardens of *Macrotermes* spp. and *Odontotermes* spp. were collected by locating and excavating their mounds. Fungus gardens of *Ancistrotermes* spp. and *Microtermes* spp. were collected by excavating soil of 50 cm depth within a 50 cm distance around termite mounds of *Macrotermes* spp. and *Odontotermes* spp. (*b*) One single fungus garden was collected from each genus nesting inside or around the mound. The number of fungal nodules on 1 g of fungus garden was counted, and diameters of 50 fungal nodules were measured.

Sampling of termite nests was conducted in three locations in Thailand, including Khon Kaen (16.4732° N, 102.8164° E), Maha Sarakham (16.1447° N, 103.0083° E), and Chiang Mai (18.7926° N, 98.9596° E). Termite mounds of fungus-growing termites, *Macrotermes* spp. and *Odontotermes* spp. in the study localities were searched and excavated to collect fungus gardens (figure 1*a*). Non-mound-building genera such as *Ancistrotermes* spp. and *Microtermes* spp. generally inhabit the soil surrounding termite mounds. To collect fungus gardens of the non-mound-building genera, soil within 50 cm of termite mounds were excavated to a depth of approximately 50 cm (figure 1*a*). For each termite mound, one single fungus garden was collected from each termite genus observed in the mound or its surrounding soil. Identification of termite species is based on the morphological descriptions and keys from Ahmad [24], Morimoto [25] and Sornnuwat *et al.* [27].

### 2.2. Quantification on size and density

Each fungus garden was subdivided into four horizontal quarters [18]. Termites on fungus gardens were collected, and minor workers were differentiated from the other castes based on their smaller head and thorax [28,29]. The top quarters of fungus gardens, which were fresh fungus garden and contain the largest number of fungal nodules [17,18], were collected and crushed into pieces for microscopic examination, using a mobile USB microscope (Model: UM12, Microlinks Technology Corp., Taiwan). For each fungus garden, diameters of 50 fungal nodules (figure 1*b*) and head widths of three minor workers were measured for quantification of their sizes, using an image analysis software M12-CAM Viewer (v. 2.507, Microlinks Technology Corp.). Head widths of the three minor workers were averaged for each fungus garden. To quantify the density of fungal nodules, 1 g of fresh fungus garden was sampled and the number of fungal nodules was manually counted under the microscope (figure 1*b*). After microscopic examination, the fungus gardens were preserved in 95% alcohol for genetic identification.

### 2.3. Genetic identification of *Termitomyces* spp.

For DNA-based identification, 5–10 fungal nodules or 250 mg of fungal gardens with visible mycelium of *Termitomyces* were collected under the microscope for subsequent DNA extraction. DNA extraction was performed using a commercially available DNA extraction kit (Plant Genomic DNA mini purification Kit, Biokit Biotechnology Incorporation, Taiwan). All procedures were performed according to the manufacturer's instructions, except for the isopropanol which was omitted from the binding buffer. This modification prevents dark pigments in termite faeces, which inhibit subsequent polymerase chain reactions (PCRs), from binding to the silica membrane along with DNA.

PCR amplification was carried out using the commercial PowerPol 2X PCR Mix (ABclonal Inc., USA), which contains proof-reading DNA polymerase, resulting in blunt-ended PCR products. ITS regions and LSU regions were first amplified with universal primers, ba9f/24R, and then 0.1 µl of the PCR product was amplified again (nested PCR) with specific primer pairs, TE1F/TE11R and TE9F/TE19R, respectively, to achieve better sensitivity and specificity. The cycling parameters were as follows: 98°C for 3 min, followed by 35 cycles of 98°C for 10 s, 62°C for 10 s, 72°C for 60 s, and one final extension step of 5 min at 72°C. PCR products were cloned into *Sma*I or *Eco*RV digested pBluescript II SK (-) vector [30]. Sequencing was conducted on an ABI PRISM 377 DNA sequencer at the Biotechnology Center of National Chung Hsing University. All obtained sequences were deposited at GenBank (electronic supplementary material, table S1).

### 2.4. Phylogenetic analyses

For phylogenetic analyses, ITS and LSU sequences of allied species were retrieved from GenBank. *Lepista nuda* CBS 247.69 was included as outgroup (electronic supplementary material, table S2). All sequences were first aligned using MAFFT (online version) [31]. The alignment was then manually inspected and poorly aligned regions were removed with Gblocks [32]. Best-fit DNA evolution models were evaluated by ModelTest-NG [33]. The maximum-likelihood tree was inferred using RAxML-NG v. 1.1.0, with Felsenstein's bootstrap (FBP) statistical support calculated from 1000 resampled datasets. The Bayesian inference (BI) tree was inferred by MrBayes v3.2.6 x64. The BI trees were based on two Markov chain Monte Carlo chains of 1 000 000 generations, with one tree sampled every 1000 generations. A consensus tree was calculated by using the last three quarters of the trees (burn-in, 750 trees in total). All analyses were performed on Ubuntu 22.04.1 (64-bit), and to ensure reproducibility, random seeds were explicitly set to 56 wherever necessary.

### 2.5. Quantification and statistical analysis

All statistical analyses were conducted using R programming language (v. 3.3.1) [34]. Differences on head width of minor workers of different termite genera, and diameter and density of fungal nodule of different *Termitomyces* clades were examined using analysis of variance (ANOVA). Correlations between head width of minor workers and diameter or density of fungal nodule were analysed using simple linear correlation models. Data used in correlation analysis were standardized (log(*n* + 1)) prior to analysis [35]. To examine the trade-off between size and density and the production limit of the fungal nodule, the distribution of volume and density of fungal nodules were fitted to an isoquant model:

L=V×D,

where *L* is the production limit of nodule volume on per gram of fungus garden (mm^3^ g^−1^), *V* is the average nodule volume (mm^3^), and *D* is the nodule density (number g^−1^). Model fitting was performed with the nonlinear least-squares method, by using the R package *nls*. *V* was calculated using the formula of an ideal sphere volume:

$$V=\;\frac{4\pi }{3}\;\times {\left(\frac{d}{2}\right)}^{3},$$

where *d* is the average diameter of the nodule.

## 3. Results

### 3.1. Diversity of *Termitomyces* and cultivar traits selected by termites

**Figure 2.**
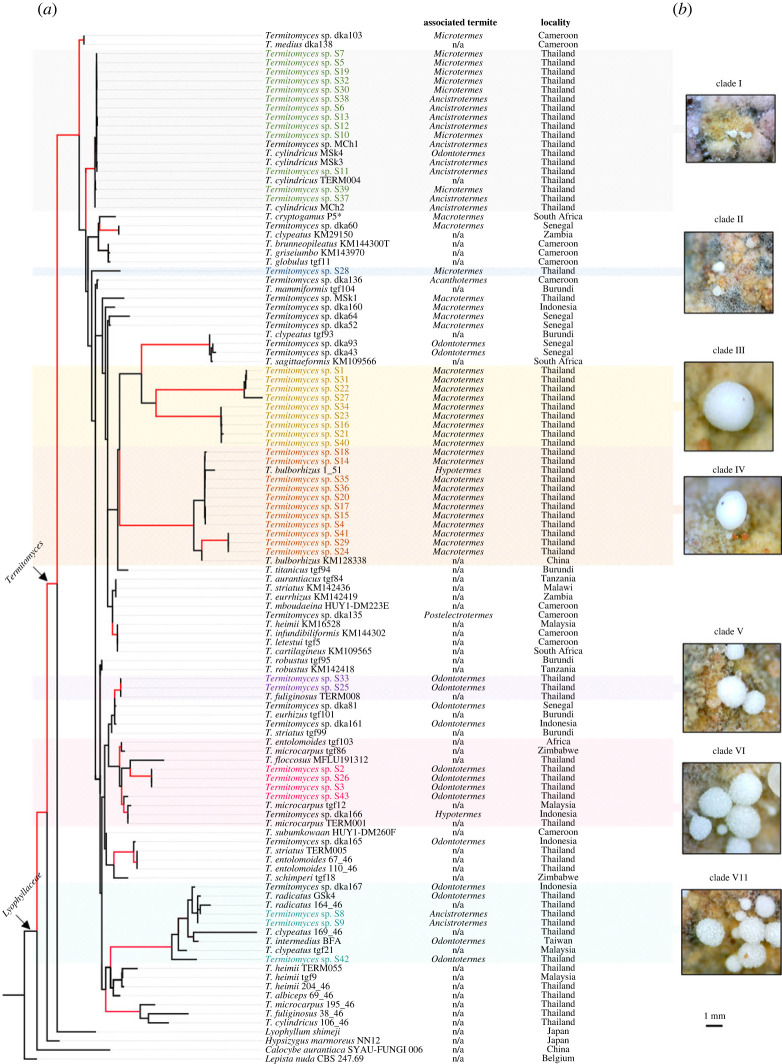
Phylogenetic positions and morphological differences of fungal nodules among clades of *Termitomyces*. (*a*) The maximum-likelihood tree inferred from the ITS and LSU regions with RAxML. Branches with FBP ≥ 70 and Bayesian posterior probabilities ≥0.9 are shown in red. Sequences generated in this study are shown in bold. (b) Different clades of *Termitomyces* produce fungal nodules with different sizes.

Our phylogenetic analyses included sequences of *Termitomyces* spp. from this and previous research. We noticed that some conspecific sequences reported by researchers were polyphyletic, for example, *Termitomyces cylindricus* 106–46 [36] and *T. cylindricus* TERM004 [37] (figure 2*a*), both of them are from Thailand and their phylogenetic positions are distant. To avoid confusion, *Termitomyces* spp. with different phylogenetic positions were named with clades in this study, instead of scientific names. Seven clades of *Termitomyces* were identified from the fungal nodules (figure 2*a*), and different clades were observed producing nodules with different sizes (figure 2*b*).

**Figure 3.**
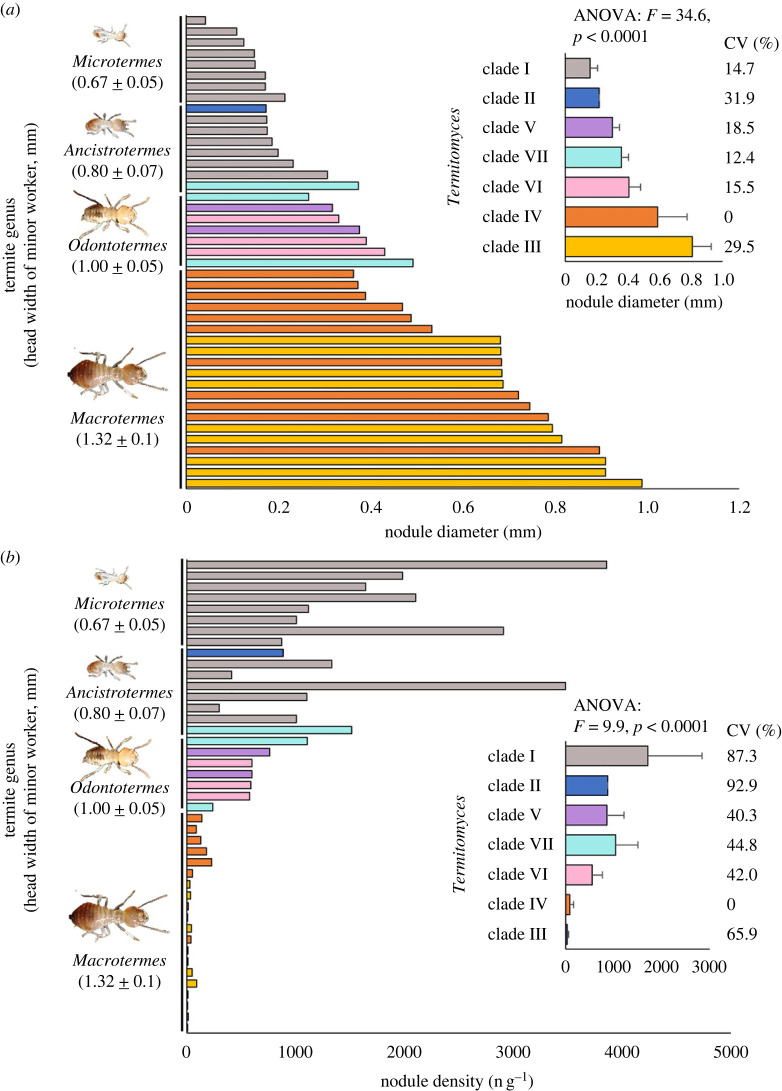
Cultivar traits of *Termitomyces* clades selected by different termite genera. (*a*) Different termite genera selectively cultivate *Termitomyces* clades that produce nodules with different diameters, and larger termites cultivate larger clades. The low coefficient of variation (CV) (12.4–31.9%) supports a specific nodule diameter was selected in each clade. (*b*) Different termite genera selectively cultivate *Termitomyces* clades that produce nodules with different density, and smaller termites cultivate clades that produce more nodules. The high CV (40.3–92.9%) supports a high plasticity of nodule density in each clade.

Diameters of a total of 2150 fungal nodules (50 nodules × 43 nests) were measured. ANOVA showed that different phylogenetic clades of *Termitomyces* produce fungal nodules with significantly different diameters (ANOVA: *F* = 34.6, *p* < 0.0001; figure 3*a*) and density (*F* = 9.9, *p* < 0.0001; figure 3*b*). The largest diameter of fungal nodules was observed in clade III, followed by clades IV, VI, VII, V, II and I (figure 3*a*). Highest density of fungal nodules was observed in clade I, followed by clades VII, II, V, VI, IV and III (figure 3*b*). The significant differences on size and density of fungal nodules among clades support that diversification of cultivar traits occurred in different clades of *Termitomyces*. The low coefficient of variation (CV) (12.4–31.9%) of nodule diameter among nests indicate a specific diameter was a stabilized trait in each clade. A high CV (40.3–92.9%) of nodule density among nests was observed.

### 3.2. Traits of fungal cultivars are associated with size of termite farmers

**Figure 4.**
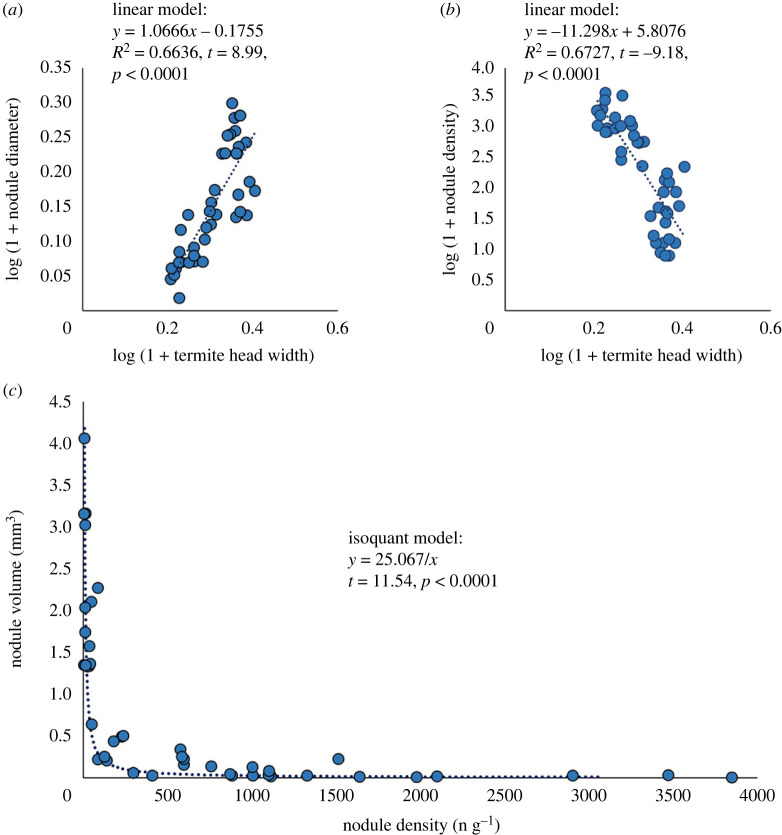
Associations between termite size and traits of their cultivars. (*a*) Head width of minor worker is positively correlated with the diameter of fungal nodules they cultivated. (*b*) Head width of minor worker is negatively correlated with the density of fungal nodules they cultivated. (*c*) Distribution of volume of fungal nodule and density fitted to isoquant model, indicates a trade-off exists in between nodule diameter and density.

Head width of minor workers of termites was significantly different among genera (ANOVA: *F* = 151.9, *p* < 0.0001; figure 3*a,b*), largest head width was observed in *Macrotermes*, followed by *Odontotermes*, *Ancistrotermes* and *Microtermes*, which demonstrates that termite farmers from different genera have different size niches. Termite genera with larger minor workers were observed cultivating *Termitomyces* that produce larger (figure 2*a*) but less numerous (figures 2*b*) fungal nodules. Correlation analysis showed that head width of minor workers was positively correlated with the diameter of fungal nodule they cultivated (figure 4*a*), and was negatively correlated with the density of fungal nodule they cultivated (figure 4*b*). These results support the idea that termite genera with larger farming castes grow larger *Termitomyces* with larger fungal nodules.

For *Termitomyces*, clades with larger fungal nodules produce less numerous nodules (figure 2*a,b*). The distribution of volume and density of fungal nodules fitted to the isoquant model (figure 4*c*; nonlinear least-squares method: *t* = 11.54, *p* < 0.0001), which indicated that volume and density of fungal nodules are negatively correlated and there is a trade-off between them. Estimation of the isoquant model indicated that volume and density of fungal nodules have a constant product of 25.067 mm^3^ g^−1^ of fungus garden.

## 4. Discussion

### 4.1. Termite-fungi size specificity and diversification of *Termitomyces*

Fungus-growing termites are widely recognized for their lack of species-specificity with their *Termitomyces* cultivars. In general, each termite species is capable of cultivating multiple *Termitomyces* species, and likewise, each *Termitomyces* species is associated with multiple termite species. However, the specificity is primarily observed at the genus level, as each termite genus primarily cultivates a group of closely related *Termitomyces* species [16,38,39]. The observed low specificity is believed to result from the horizontal transmission mode used by fungus-growing termites [16,38]. In this study, we corroborate the genus-level specificity between fungus-growing termites and *Termitomyces* spp. Furthermore, our findings indicate that specificity is linked to size, whereby larger termites cultivate *Termitomyces* spp. that produce larger nodules.

Our phylogenetic analysis reveals that each clade of *Termitomyces* tends to be cultivated by a group of termite genera with comparable body sizes. For instance, clade I, which exhibits the smallest nodules, is cultivated by small to medium-sized genera, namely *Microtermes*, *Ancistrotermes*, and *Odontotermes* (figure 2). Clade VII, which produces medium sized nodules, is cultivated by *Ancistrotermes* and *Odontotermes*, but is unavailable for the smallest termite, *Microtermes* (figure 2). Clade IV, which bears the second largest nodules, is tended to by medium to large-sized genera, *Hypotermes* and *Macrotermes* (figure 2). While measurements of minor workers of *Hypotermes* were unavailable in this study, the sizes of its soldier and major worker castes are estimated to lie between those of *Odontotermes* and *Macrotermes* [24]. Our findings provide novel insights into the mechanisms that govern the specificity of insect-fungi associations in a horizontal transmission mode.

The size specificity of termite-fungi could serve as a mechanism contributing to niche partitioning and species diversification in *Termitomyces*. Mitochondrial genome sequences reveal that *Ancistrotermes* and *Microtermes* are sister groups of *Macrotermes* and *Odontotermes*, respectively, with *Macrotermes* being the sister group of *Odontotermes* [26]. This phylogenetic relationship implied that the niche of small *Termitomyces* strains is available prior to larger ones. We propose an evolutionary scenario for coevolution between termites and *Termitomyces* involving at least two stages: (i) small-sized fungus-growing termites and small-sized fungal nodules are selected from their common ancestors, and (ii) larger fungus-growing termites, *Macrotermes* and *Odontotermes*, as well as their larger cultivars are selected from their small-sized ancestors. The phylogenetic relationship between termites and fungi indicates that the coexistence of a varied group of fungus-growing termites has reduced the selective pressure on *Termitomyces* to invest in a specific nodule size, thus facilitating fungal diversification. Mechanisms underlying the termite-fungi size specificity are considered related to differential modes of transmission at different colony development stages, which are discussed in the following paragraphs.

### 4.2. Environment-nest transmission and the availability of *Termitomyces* strains

It is unlikely that the observed size specificity between *Termitomyces* and fungus-growing termites is attributable to differences in the availability of *Termitomyces* spores in habitats inhabited by different termite genera, for two reasons. Firstly, *Termitomyces* spp. exhibit horizontal transmission, whereby they release their sexual spores to the environment through fruiting bodies [40]. Although vertical transmission is also observed in one species of *Macrotermes* and in the genus *Microtermes* [15], vertical transmission in *Microtermes* does not restrict the exchange of symbionts [41], which indicates that horizontal transmission is still available in *Microtermes*. Therefore, the distribution of *Termitomyces* spores is not limited to specific habitats or termite genera. Secondly, all termite genera are sympatric, coexisting within the same locality (electronic supplementary material, table S1) or even sharing the same mound (electronic supplementary material, figure S1*a*), thereby suggesting that different termite genera share a common pool of spores in the environment.

**Figure 5.**
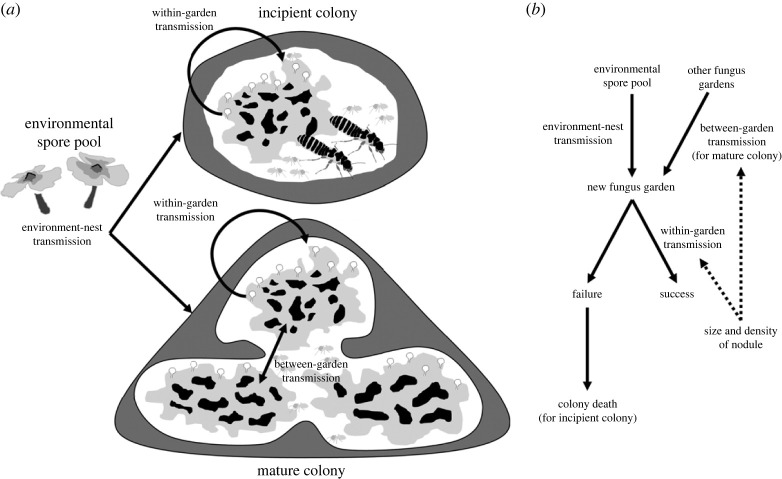
The modes of transmission of fungal spores in termites and the mechanism of maintaining a specific size of nodules. (*a*) Termites are assumed to gather spores of *Termitomyces* from a shared pool in the environment, and they transmit them to the fungus garden. This transmission occurs both within the same garden (within-garden transmission) and between different gardens (between-garden transmission), which happens in mature colonies. (*b*) The rate of transmission is affected by size. If the size of the fungal strain mismatches with termite size, incipient colonies may fail. On the other hand, between-garden transmission ensures monoculture in mature colonies and maintains the dominance of strains with matched size.

If spores are available in the environment, horizontal transmission of fungal spores cannot be completely avoided, even in cases where the fungi are acquired through vertical transmission, such as in ambrosia beetles [42,43]. Based on this assumption, it is possible that termite nests could be inoculated with multiple strains of *Termitomyces* that produce fungal nodules of varying sizes at any given stage of colony development (figure 5). Earlier studies have suggested that termites engage in monoculture of fungi within a closed system, as genetic tools have failed to detect secondary *Termitomyces* strains in termite nests [44–46]. However, it is essential to recognize that the failure to detect secondary *Termitomyces* does not necessarily imply their absence. The co-existence of multiple strains within a colony is detected by the genetic analysis of fungus gardens in *Odontotermes* and *Macrotermes* [47], which support the idea that horizontal transmission of *Termitomyces* occurs in mature colonies.

### 4.3. Selection of matched pairs in colony-founding stage

Since fungal spores can be acquired horizontally [15,16], it is possible for incipient termite colonies to obtain and cultivate a diverse range of fungal cultivars at the same time (figure 5). In the event that the termites are unable to acquire compatible cultivar, such as when a large termite is paired with a small nodule or a small termite is paired with a large nodule, the colony is at risk of failure (figure 5*b*). The nutrition requirements of the termites may be a factor that drives the selection against such mismatched size of termite-fungi combinations. For instance, the volume of a single nodule produced by *Termitomyces* clade III is approximately equivalent to the combined volume of approximately 166 nodules produced by clade I. This observation implies that termites would have to collect 166 nodules of clade I to obtain the same amount of nutrition obtained from a single nodule produced by clade III. In cases where small and large termite farmers coexist in sympatric conditions, it is conceivable that they could obtain spores of strains cultivated by each other. However, it is also predictable that the harvest process would be significantly inefficient if clade I were to be transferred and dominate the fungus gardens of a large fungus-grower such as *Macrotermes*. Furthermore, if clade III were to be transferred and dominate the fungus gardens of *Microtermes*, the resultant fungal nodules would be excessively large for the workers to consume.

Notably, incipient colonies of fungus-growing termites typically contain a single fungus garden. Under such conditions, the dominant strain of *Termitomyces* is expected to produce more spores and is likely to maintain its dominance within the fungus garden [48]. In addition, the study conducted by Aanen *et al.* [44] suggests that fusions between genetically related *Termitomyces* strains may result in higher spore production, thereby enhancing monoculture within the fungus garden. In this context, only one single *Termitomyces* strain that matched the size of termite would be maintained on the fungus garden.

### 4.4. Maintenance of the matched cultivar in mature colonies

Termite colonies have a lifespan of over 10 years [49], and termites continuously forage for food, making it unlikely to prevent the introduction of additional *Termitomyces* strains. The entry of a new *Termitomyces* strain into a termite nest can lead to competition between it and the pre-existing strain. However, the spores' transmission between fungus gardens through termite workers may influence this competition (as illustrated in figure 5*b*). Our isoquant model indicates that different strains exhibit a similar production rate of nodule mass per gram of fungus garden, suggesting that spore production is most likely related to the extent of the fungus garden they occupy. Hence, it is foreseeable that the dominant resident strain, which occupies most of the fungus gardens in the nest, would produce more spores and outcompete the introduced *Termitomyces* strain in newly constructed fungus gardens. As a result, any potential mismatches between cultivars would be prevented from arising within the termite nest.

### 4.5. Size and density of fungal spore as a tool to assess diversity of *Termitomyces*

*Termitomyces* spp. is a large group of fungi consisting of over one hundred species [50]. While most of the *Termitomyces* life cycle occurs in the asexual phase, confined to the termite nest, species identification is typically based on the morphology of structures in the sexual phase, namely the basidiocarp and spores [37]. These are collected from outside the termite nest during specific seasons, and are unable to link to the asexual phase. Without the morphological descriptions, the identification of *Termitomyces* in the asexual stage currently relies on genetic databases. In this study, we have primarily demonstrated that different *Termitomyces* strains produce fungal nodules with distinct sizes and densities (as illustrated in figures 2 and 3). Although the density of nodules may be influenced by termite harvesting activity, the significant variations observed among different clades can still serve as a distinguishing characteristic. This finding could serve as a valuable tool for identifying *Termitomyces* species within a termite nest.

## 5. Conclusion

In conclusion, the present study has demonstrated that a size-specific relationship between *Termitomyces* and fungus-growing termites exists. Specifically, larger termite genera were found to cultivate *Termitomyces* with larger but fewer nodules. Moreover, we found a trade-off between size and density, which suggests that the spore production of a given strain within the nest is dependent on the extent of the fungus garden that it occupies. We propose that the size specificity is maintained through two mechanisms: (i) termites are unable to establish colonies with a mismatched size of *Termitomyces*, and (ii) the dominance of an established strain prevents the emergence of introduced strains of *Termitomyces*. Artificial inoculation of *Termitomyces* strains on laboratory colonies is essential for testing these hypotheses.

## Data Availability

Morphological measurements of termites and fungal nodules are available in the text. DNA sequences of *Termitomyces* spp. are deposited in GenBank (electronic supplementary material, S2 [51]).
